# Prof. James Syme on Iridectomy

**Published:** 1864-04

**Authors:** 


PROF. JAMES SYME ON IRIDECTOMY.—Sir:—As you ask my opinion of iridectomy, I have no hesitation in saying that it has always seemed to me an entire delusion accepted for the cure of blindness, on the same principle which leads drowning men to catch at straws. Glaucoma has been regarded as so hopeless a disease, that it was peculiarly well suited for the proposal of an operation which promised merely to afford some chance of relief. Such being its modest profession, the destructive inflammation, lenticular opacity, and collapse of the eye-ball, which too frequently result from opening the cornea and cutting out a portion of the iris, were not held to counterbalance the benefit claimed by patients so fortunate as to escape these dangers. But this alleged benefit, from what has come under my observation, does not appear to be at all different from that which every one laboring under incurable deafness may believe for a time he has received from the use of remedial means, whatever they may have been. The truth is, that any man who has paid money, and suffered pain, does not like to confess that his object in doing so has not been accomplished; while his attention and imagination being at the same time excited, he is apt to regard the feeblest glimmer of light, or the faintest perception of sound, as a symptom of improvement. Iridectomy will, therefore, I trust, soon disappear, not only from surgical practice, but from surgical language.

I am, &c., JAMES SYME.

—*British Medical Journal.*

*Messrs. Editors:*—Our humorous friend, the *London Punch*, had, some time ago, a picture called "Wholesome Prejudice." A heavy-built old English gentleman is sitting at a table, on which stands his glass of "port," with which and indignation his face is flushed as he gives vent to the following, in answer probably to some inquiry of a fellow traveler:—"Railroads, Sir?

I hate railroads, and I shall be very glad when they're done away with, and we've got the coaches again."

This was vividly brought back to my mind by seeing the answer of Prof. James Syme to the *British Medical Journal*, October 24, 1863, in regard to "iridectomy." Certainly we all would agree that if iridectomy in Prof. Syme's hands "*too often results in destructive inflammation, lenticular opacity, and collapse of the eye-ball*," the sooner it "disappears not only from" *his* "surgical practice, but from" *his* "surgical language,' the better.

Respectfully, B. JOY JEFFRIES.

15 Chestnut St., Feb. 19th, 1864.

—*Boston Medical Journal.*

